# The evolution of stress urinary incontinence treatment techniques of the last three decades

**DOI:** 10.1590/S1677-5538.IBJU.2021.0646.1

**Published:** 2022-07-19

**Authors:** Cassio Luis Zanettini Riccetto

**Affiliations:** 1 Divisão de Urologia Feminina UNICAMP Campinas SP Brasil Divisão de Urologia Feminina – Universidade de Campinas Faculdade de Ciências Médicas – UNICAMP, Campinas, SP, Brasil

## COMMENT

The authors retrospectively studied a database of 221 patients who underwent correction of stress urinary incontinence (SUI) through the implantation of a SAFYRE VS retropubic sling (96 women) or a homemade polypropylene retropubic sling - HMS (125 patients) between March 2005 and December 2007, comprising a median follow-up of 78.47 (± 38.69) months ( 1 ). The evaluation included a telephone call made by a blinded trained researcher for those patients who had completed at least one year of surgery. The HMS was made of a 75g/m^2^, 15mm-wide polypropylene mesh attached with polyglycolic acid sutures at its edges. Both HMS and SAFYRE VS groups presented significant improvements on International consensus on Incontinence – Urinary Incontinence Short Form questionnaire (ICIQ-UI SF) and there were no differences in satisfaction, subjective cure rates, ICIQ-UI SF, or complications between groups, but a significantly higher frequency of patients of SAFYERE VS group required indwelling urinary catheter over 24 hours ( 2.4% vs. 8.3%, p=0.061) as well as a higher frequency of bladder injury was observed in the SAFYRE VS group (0% vs. 4.2%, p=0.034).

In the present study ( 1 ), the use of the SAFYRE VS was not advised for patients with severe or recurrent SUI or those with expected need of postoperative readjustment that are the primary population for which readjstuble sinthetic slings have been currently proposed ( 2 , 3 ). In fact, authors disclosured that the allocation of patients for HMS or SAFYRE VS implant was exclusively conditioned to their availability at the time of surgery. Furthermore, no significant sociodemographic or clinical differences were detected between patients in both groups, which allowed for reasonable data comparison despite the retrospective and non randomized study design.

In fact, there are few publications focused on both types of suburethral slings which were studied in the current series. The SAFYRE VS sling kit developed in Latin America, and together with REEMEX readjustable System (Neomedic Int, Spain) correspond to the only two slings that propose to allow an easy postoperative readjustment feature ( 4 ). However, publications on long-term follow-up are rare for both devices so the present series is a good reference on the performance of SAFYRE VS in longer follow-up periods than previously published (refer to article’s references).

Publications about homemade polypropylene slings are even rarer and much more difficult to evaluate, due to the biomechanical differences and the wide range of of the meshe’s size resulted from the surgeon’s tailoring. In addition, detailed descriptions of the procedures used for the primary adjustment and sling fixation are often missed in the publications ( 5 ), leaving no answer as to how it should be performed, i.e., if similar to the adjustment of a classic aponeurotic sling or as the same manner as used for polypropylene midurethral slings sets.

Contrary to the results presented in this article, it could be assumed that the rate of prolonged urethral catheterization would possibly be higher in the HMS group, either because of the difficulties inherent in fitting a non-industrialized sling, or because of the authors’ option of using a slightly wider sling (15mm) than the available minimally invasive midurethral slings sets. The authors attributed their findings to the design of the SAFYRE VS silicone columns. In this sense, we could hypothesize that, according to the authors, the lower friction of the silicone columns against the host tissues, or even an eventual elastic effect of the silicone columns could add a risk of excessive traction by the surgeon and so some obstruction effect. In this sense, it should be reasonable to advise those that intent to implant a SAFYRE VS sling that additional care should be taken when adjusting this kind of sling. Additionally, one should consider that it seems that tightening the SAFYRE VS should be simpler than loosening it.

In conclusion, the publication of such article rescues interesting aspects the evolution of SUI treatment techniquesof the last three decades. We congratulate the authors for their willingness to share their results and thoughs.
